# Polymorphisms in four microRNAs and risk of oral squamous cell cancer: a meta-analysis

**DOI:** 10.18632/oncotarget.24211

**Published:** 2018-01-12

**Authors:** Junfeng Zeng, Xiaowei Yi, Hao Liu, Yi Yang, Yuchen Duan, Hua Chen

**Affiliations:** ^1^ Department of Orthopedics, West China Hospital, Sichuan University, Chengdu, 610041, China; ^2^ State Key Laboratory of Oral Diseases, National Clinical Research Center for Oral Diseases, West China Hospital of Stomatology, Sichuan University, Chengdu,610041, China

**Keywords:** microRNAs, polymorphism, oral squamous cell cancer (OSCC), cancer biomarkers, meta-analysis

## Abstract

**Objectives:**

Single nucleotide polymorphisms in microRNAs (microRNA-196a2 rs11614913, microRNA-146a rs2910164, microRNA-149 rs2292832 and microRNA-499 rs3746444) have been inconsistently associated with risk for oral squamous cell cancer (OSCC). This meta-analysis aimed to assess the correlation between microRNA polymorphisms and susceptibility to OSCC.

**Materials and Methods:**

Free words were used to search for the relevant studies without language limitations in electronic databases including PubMed, Embase, Web of Science and SCOPUS through June 15, 2017. Odds ratios (ORs) were calculated to investigate the effects of microRNA polymorphisms on oral cancer risk.

**Results:**

Eleven studies were included. Analysis under the recessive model of microRNA-146a (CC vs GG+CG) showed significant differences (ORs = 0.874, P = 0.041). The G allele and the GG genotype of microRNA-499 were associated with OSCC risk (ORs >1, *P* < 0.05). MicroRNA-196a2 rs11614913 and microRNA-149 polymorphisms appeared to have no relationship with OSCC risk (*P* > 0.05). In the sensitivity analysis, there was a significant association between the TT genotype of microRNA-196a2 and OSCC risk (TT vs TC + CC, ORs < 1, *P* < 0.05).

**Conclusions:**

There may be no significant relationship between microRNA-149 polymorphisms and OSCC risk, and the CC genotype of microRNA-146a may have protective effects against oral cancer. However, the G allele and the GG genotype of microRNA-499 may increase OSCC risk.

## INTRODUCTION

The Incidence of oral cancer cases has been steadily increasing, due to population growth and changes in age structure [1]. Globally, the incidence of oral cancer has been estimated to increase by 62% through 2035 [2]. Oral squamous cell cancer (OSCC) is the most common malignancy of oral cancers, accounting for approximately 90% of all oral cancers [3]. Numerous studies have aimed to explore and uncover risk factors for developing OSCC. Genetic variation was highlighted in previous studies as a potential risk factor [4, 5].

MicroRNAs (miRNAs), which are endogenous, small non-coding RNAs, are critical to the regulation of physiology and disease progression, including oral cancers [6]. MiRNAs can induce messenger RNA (mRNA) cleavage or repress translation by targeting the 3’-untranslated region (UTR) of mRNA [7]. Consequently, dysregulated protein expression associated with single nucleotide polymorphisms (SNPs) in miRNAs can alter OSCC etiology.

MiRNAs exhibit remarkable tissue specificity [8], and miRNA biomarkers for OSCC may differ from those of other cancers. Several aberrantly expressed miRNA variants have been observed in OSCC patients, including microRNA-196a2 [9–16], microRNA-146a [9, 10, 12, 14, 17, 18], microRNA-149 [10, 12, 15] and microRNA-499 [9, 10, 19], among others. However, no consensus has been reached regarding whether and to what extent miRNAs could influence susceptibility to OSCC.

Abnormal microRNA polymorphisms in OSCC warrant more research to provide a basis for diagnosis, therapy and screening for the high-risk OSCC population. Advances in our efforts to detect the role of miRNAs in genetic susceptibility to OSCC may further elucidate their role in regulating OSCC risk and may identify certain miRNAs as oncogenic biomarkers. Therefore, the purpose of this study was to review the literature for evidence of correlations between certain microRNA polymorphisms and the risk of oral squamous cell cancer.

## RESULTS

### Literature search and study inclusion

**Figure 1:**
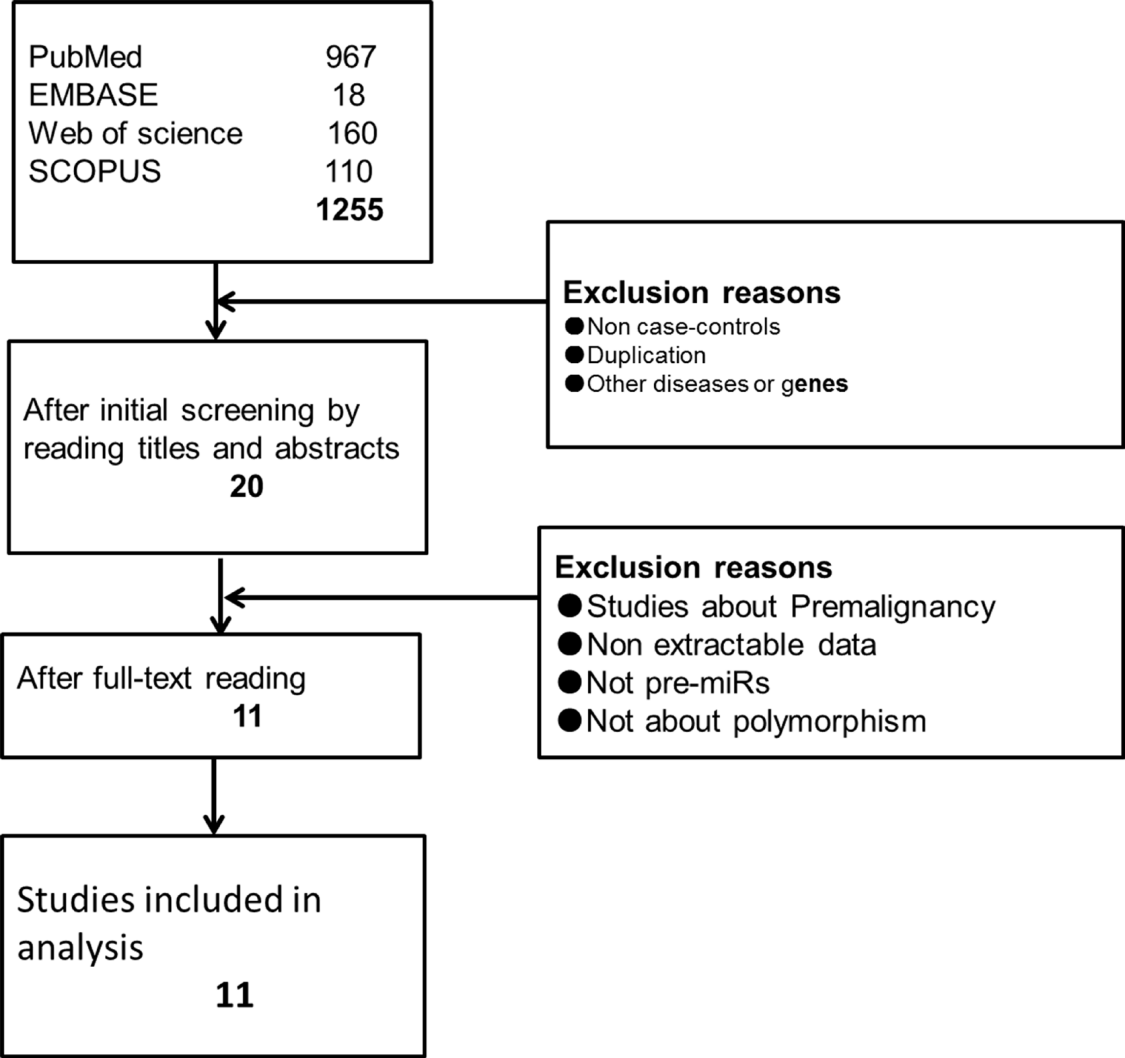
Flow diagram of screening studies

The initial electronic database searches of PubMed, Embase, Web of science and SCOPUS yielded 967, 18, 160, and 110 potentially studies, respectively. After reading the titles and abstracts of those studies, 20 articles were chosen for full-text reading, and their references were screened. Finally, 11 eligible studies on SNPs in miRNAs were included after the unqualified studies were discarded (Figure 1). Following that assessment, a data extraction form was constructed for the characteristics of the chosen studies (Table 1), and the data from the 11 included studies were extracted and synthesized. Of the 11 studies, 3 studies were given a score of 5 and recognized as high risk. In contrast, 3 high-quality trials were given a score of 7. The remaining 5 studies had a moderate risk of bias (Table 2).

**Table 1:** Characteristics of the included studies on microRNA polymorphisms and risk of oral squamous cell cancer

Reference	Country	Source of control	Case/Control	Genotype distribution										
														P for HWE
				Case				Control						
microRNA-196a2 rs11614913 C > T															
				C	T	CC	TC	TT	C	T	CC	TC	TT	
Zhang E, 2017	China	Hospital-based	340/340	311	369	71	169	100	331	349	88	155	97	0.106
Miao L, 2016	China	Hospital-based	462/1550	452	472	112	228	122	1339	1761	292	755	503	0.796
Sushma PS，2015	India	Not mentioned	100/102	54	146	22	10	68	27	177	6	15	81	0.00028
Roy R, 2014	India	Hospital-based	451/448	623	279	218	187	46	652	244	242	168	38	0.25
Liu CJ, 2013	Taiwan	Not mentioned	315/92	275	355	64	147	104	88	96	26	36	30	0.04
Chu YH, 2012	Taiwan	Hospital-based	470/425	391	549	57	277	136	380	470	87	206	132	0.68
microRNA-146a rs2910164 G > C															
				G	C	GG	CG	CC	G	C	GG	CG	CC	
Zhang E, 2017	China	Hospital-based	340/340	178	502	27	124	189	152	528	19	114	207	0.53
Miao L, 2016	China	Hospital-based	462/1548	536	388	154	228	80	1767	1329	497	773	278	0.46
Palmieri A, 2014	Italy	Population-based	337/1176	515	159	197	121	19	1730	622	647	436	93	0.10
Chu YH, 2012	Taiwan	Hospital-based	470/425	350	590	54	242	174	304	546	54	196	175	0.93
Chen CH, 2016	Taiwan	Hospital-based	512/668	383	641	71	241	200	499	837	103	293	272	0.10
microRNA-149 rs2292832 C > T															
				T	C	TT	TC	CC	T	C	TT	TC	CC	
Miao L, 2016	China	Hospital-based	461/1548	645	277	226	193	42	2099	997	726	647	175	0.092
Sushma PS, 2015	India	Not mentioned	100/102	34	166	6	22	72	12	192	0	12	90	0.52
Chu YH, 2012	Taiwan	Hospital-based	470/425	778	162	345	88	37	714	136	315	84	26	4.84
microRNA-499 rs3746444 A > G															
				G	A	GG	GA	AA	G	A	GG	GA	AA	
Zhang E, 2017	China	Hospital-based	340/340	180	500	31	118	191	135	545	12	111	217	0.633
Hou YY, 2015	China, Taiwan	Hospital-based	155/204	49	261	5	39	111	53	355	1	51	152	0.13
Chu YH, 2012	China, Taiwan	Hospital-based	470/425	143	797	12	119	339	72	778	3	66	356	0.97

HWE: Hardy-Weinberg equilibrium

**Table 2:** NOS scores of included studies

Author (Ref)	Year	Case	Control		Comparability		Exposure				Score
		Definition	Represent ativeness	Selection	Definition	Important factors	Other factors	Ascertain -ment	Method	Non-response rate	
Zhang E	2017	★	★	☆	★	★	★	☆	★	☆	6
Miao L	2016	★	★	★	★	★	★	☆	★	☆	7
Sushma PS	2015	☆	★	☆	★	★	★	☆	★	☆	5
Hou, YY	2015	★	★	☆	★	★	★	☆	★	☆	6
Roy, R	2014	☆	★	☆	★	★	★	☆	★	☆	5
Liu CJ	2013	★	★	☆	★	★	★	☆	★	☆	5
Song X	2013	★	★	☆	★	★	★	☆	★	★	7
Chu YH	2012	☆	★	☆	★	★	★	☆	★	★	6
Palmieri a	2014	★	★	☆	★	★	★	☆	★	☆	6
Chen CH	2016	★	★	☆	★	★	★	☆	★	☆	6
Christensen BC	2010	★	★	★	★	★	★	☆	★	☆	7

NOS: Newcastle-Ottawa Scale

### Association between the microRNA-196a2 rs11614913 polymorphism and risk of OSCC (Table 3)

**Table 3:** Summary of tests for association between polymorphisms in four microRNAs and OSCC risk, of publication bias, and of included study number, under all genetic models of four miRNAs

Comparison	Test of association			Publication bias			Number	
	ORs	95% CIs	*P*	PEgger	PBegg		Cases	Controls
microRNA-196a2 rs11614913 C > T								
T vs C	0.984	0.903∼1.073	0.719	0.803	0.707		4276	5914
TT vs CC	0.974	0.817∼1.161	0.766	0.992	1.000		952	1566
TC vs CC	1.159	0.999∼1.345	0.051	0.838	0.707		1759	2076
TT + TC vs CC	1.105	0.937∼1.10	0.710	0.926	0.902		2732	3847
TT vs TC + CC	0.877	0.763∼1.007	0.063	0.696	0.707		1823	2865
microRNA-146a rs2910164 G > C								
G vs C	0.946	0.877∼1.021	0.153	0.259	0.452		4936	10298
GG vs CC	0.921	0.776∼1.092	0.344	0.258	0.707		1386	2988
GC vs CC	1.008	0.890∼1.141	0.900	0.818	0.707		1785	4067
GG + GC vs CC	1.000	0.896∼1.117	0.996	0.951	0.764		2793	5484
GG vs CC + GC	0.874	0.768∼0.994	0.041	0.100	0.221		2468	5149
microRNA-149 rs2292832 C > T								
T vs C	1.098	0.963∼1.252	0.161	0.494	1.000		2062	4150
TT vs CC	1.172	0.875∼1.569	0.287	0.636	1.000		728	1332
TC vs CC	1.195	0.896∼1.593	0.225	0.850	1.000		454	1034
TT + TC vs CC	1.128	0.923∼1.389	0.238	0.480	0.734		1356	2410
TT vs TC + CC	1.070	0.903∼1.267	0.437	0.396	1.000		1031	2075
microRNA-499 rs3746444 A > G								
G vs A	1.570	1.318∼1.871	0.000	0.802	1.000		1930	1938
GG vs AA	3.430	1.914∼6.146	0.000	0.023	0.296		689	741
GA vs AA	1.407	1.142∼1.733	0.001	0.668	1.000		917	953
GG + GA vs AA	1.484	1.253∼1.758	0.000	0.621	0.734		1290	1304
GG vs GA + AA	3.165	1.777∼5.637	0.000	0.061		0.296	965	969

PEgger: *P* value for Egger’s test. PBegg: *P* value for Begg’s test. No significant publication bias is observed if PEgger > 0.05 and PBegg > 0.05.

**Figure 2:**
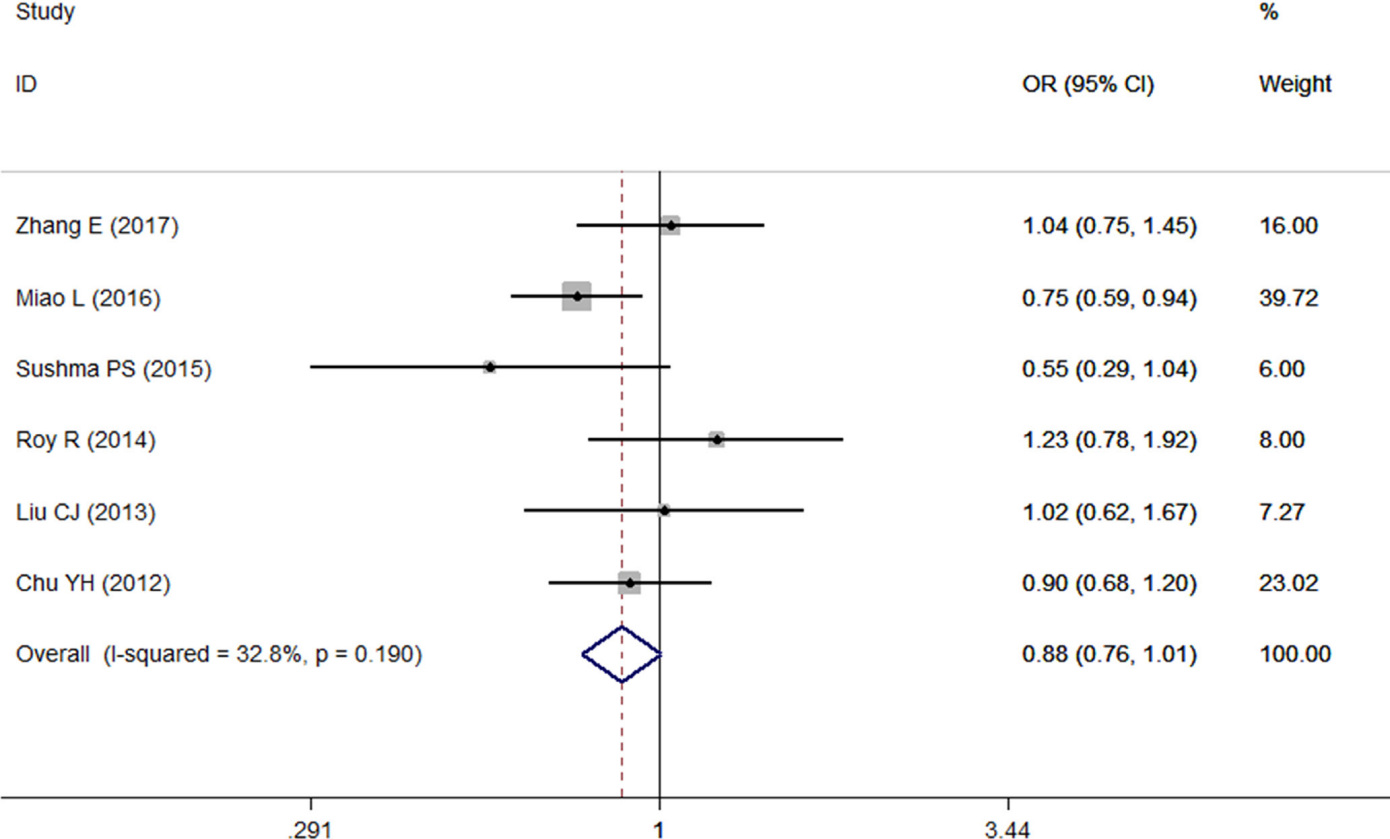
Forest plot of the relationship between the TT genotype of microRNA-196a2 and OSCC risk under TT vs TC+CC

A total of 8 studies [9–16] on the dominant model and 6 studies [9–13, 15] on the other 4 genetic models were involved in this meta-analysis. There was no significant association between the microRNA-196a2 polymorphisms and risk of OSCC under any genetic model tested (*P* > 0.05 for all models). The ORs in all 5 models were close to 1, which may suggest that the microRNA-196a2 *rs11614913* polymorphism has a very low effect on OSCC risk. However, the sensitivity analysis, which was conducted by eliminating one study at a time [9, 11, 13], indicated that the TT genotype of microRNA-196a2 *rs11614913* decreased OSCC risk *(TT* vs *TC+CC, I*^***2***^ *< 50%, OR < 1, P < 0.05)* (Figure 2).

### Association between the microRNA-146a rs2910164 polymorphism and risk of OSCC (Table 3)

**Figure 3:**
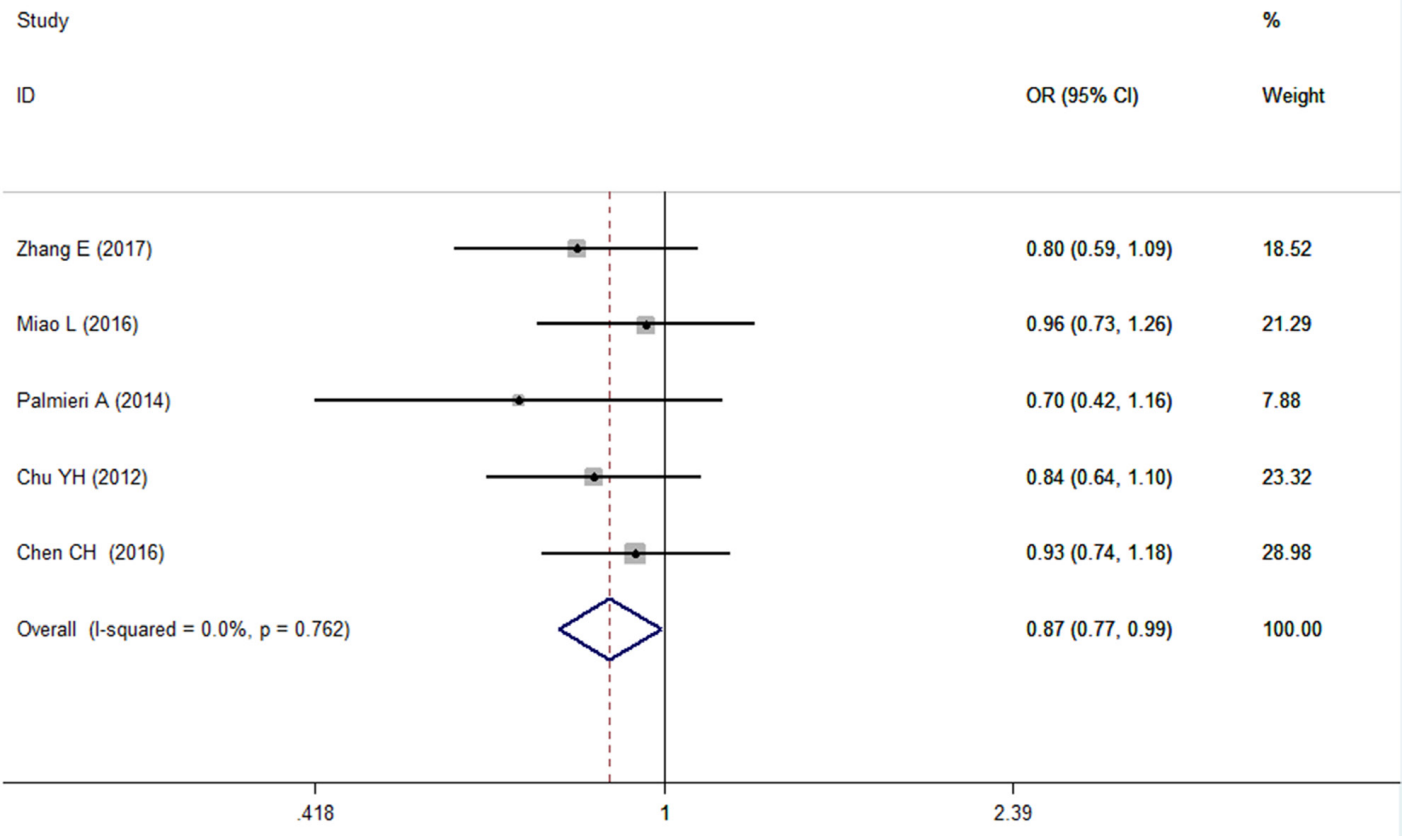
Forest plot of the relationship between the CC genotype of microRNA-146a and OSCC risk under CC vs GG+CG

A pooled analysis of 6 studies [9, 10, 12, 14, 17, 18] involving 2323 cases and 5059 controls showed no significant changes under any genetic model, except for the recessive model *(CC* vs *GG + CG, I*^***2***^ *= 0%, OR = 0.874, 95% CI = 0.768∼0.994, P = 0.041)* (Figure 3). The genotype allocation of the microRNA-146a rs2910164 polymorphism was found to be in Hardy–Weinberg equilibrium (HWE) in all included comparisons (*P*_HWE_ > 0.05 for all models). Heterogeneity was not found in any of the genetic models (I^2^ = 0% for all). The high level of agreement among the included studies provided powerful evidence that there is a strong negative correlation between the CC genotype of microRNA-146a rs2910164 and OSCC risk.

### Association between the microRNA-149 rs2292832 polymorphism and risk of OSCC (Table 3)

For microRNA-149 rs2292832, 3 studies [10, 12, 15] were eligible, involving 1356 cases and 2410 controls. Although the ORs in all genetic models were greater than 1, no significant association was observed in any of the 5 genetic models. However, these studies showed apparent heterogeneity (I^2^ ≥ 50%). Therefore, further well-designed studies investigating the relationship between the microRNA-149 rs2292832 polymorphism and risk of OSCC are warranted.

### Association between the microRNA-499 rs3746444 polymorphism and risk of OSCC (Table 3)

**Figure 4:**
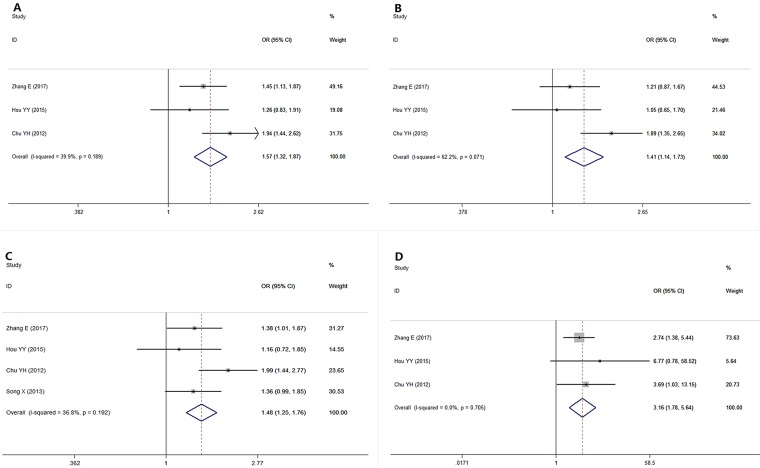
Forest plot showing a significant association between the microRNA-499 polymorphism and OSCC risk under various genetic models (**A**) Allele model: G vs A, (**B**) Codominant model: GA vs AA, (**C**) Dominant model, GG+GA vs AA (**D**) Recessive model: GG vs GA+AA.

As indicated in Table 3 and Figure 4, a pooled analysis of 3 studies [9, 10, 19] showed a significant association between the microRNA-499 rs3746444 polymorphism and risk for OSCC under the allele model (G vs A, I^2^ = 39.9%, OR = 1.57, 95% CI = 1.318∼1.871, *P* = 0.000), the additive model (GG vs AA, I^2^ = 0%, OR = 3.430, 95% CI = 1.914∼6.146, *P* = 0.000), the codominant model (GA vs AA, I^2^ = 62.2%, OR = 1.407, 95% CI = 1.142∼1.733, *P* = 0.001), the dominant model (GG+GA vs AA, I^2^ = 36.8%, OR = 1.484, 95% CI = 1.253∼1.758, *P* = 0.000) and the recessive model (GG vs GA+AA, I^2^ = 0%, OR = 3.165, 95% CI = 1.777∼5.637, *P* < 0.00001). The high ORs in all genetic models (ORs > 1) indicate that the G allele and the GG genotype of microRNA-499 rs3746444 increase the risk for oral cancer.

### Publication bias and sensitivity analysis

**Figure 5:**
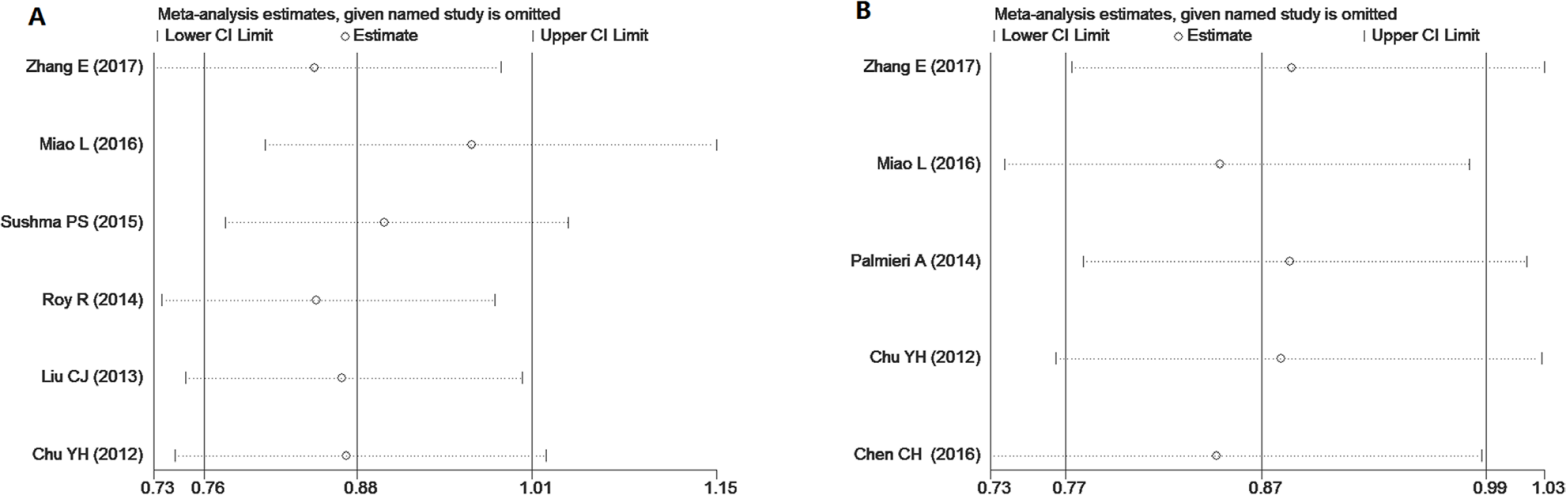
Sensitivity analysis conducted by sequentially eliminating one study at a time to examine the relationship between the TT genotype of miR-196a2 (**A**), the CC genotype of miR-146a (**B**) and OSCC risk.

To confirm the outcome of our analyses, we conducted a sensitivity analysis by sequentially eliminating one study at a time. The sensitivity analysis revealed that certain studies significantly influenced the correlation between microRNA polymorphism and risk of OSCC. The TT genotype of microRNA-196a2 rs11614913 was found to potentially decrease OSCC risk *(TT* vs *TC+CC, I*^***2***^ *< 50%, OR < 1, P* < 0.05) when three studies were excluded [9, 11, 13] (Figure 5). From the illustrated data on microRNA-196a2 in those three studies, the authors confused minor allele frequency (MAF) [9], and the genetic distribution of the controls was not in HWE [11]. For the recessive model of microRNA-146a, three studies [9, 10, 17] influenced the statistical significance of the correlation between polymorphism and risk of OSCC (Figure 5). We surmise that the small number of studies included in our meta-analysis may contribute to the influence of the aforementioned studies; if more studies had been included, the influence of any one study would be decreased.

We performed Egger’s test and Begg’s test to determine publication bias (P_Egger_> 0.05, P_Begg_> 0.05) using Stata version 14.0 software (StataCorp LP, College Station, TX, USA). No significant publication bias was observed, except in the case of the additive model of microRNA-499 (Table 3).

## DISCUSSION

Numerous studies have been conducted to investigate whether miRNAs affect susceptibility to OSCC, and the results have been controversial and inconsistent. This is the first meta-analysis conducted to assess the association between selected miRNAs and OSCC risk. Data from 8 and 3 studies indicated that microRNA-196a2 rs11614913 and microRNA-149 rs2292832 polymorphisms, respectively, have no significant effect on OSCC risk. However, the sensitivity analysis suggested that the TT genotype of microRNA-196a2 may decrease OSCC risk when one study was excluded. We also found that the GG genotype of microRNA-146a rs2910164 may negatively regulate OSCC risk. The pooled analysis of 3 studies showed that there may be a significant association between the G allele and the GG genotype of the microRNA-499 rs3746444 polymorphism and risk for OSCC.

OSCC is one of the most challenging and aggressive cancers, and it remains difficult to detect. MiRNAs, which are regulators of biological processes, regulate the pathogenesis of oral cancer and are thought to be potential biomarkers in cancer diagnosis, based upon their aberrant expression and single nucleotide polymorphisms [20]. Novel therapeutic techniques can also target miRNAs to treat OSCC. In a systematic review, the 4 miRNAs discussed in this report (microRNA-196a2 rs11614913, microRNA-146a rs2910164, microRNA-149 rs2292832 and microRNA-499 rs3746444) were found to potentially contribute to the genesis, progression and prognosis of OSCC [21]. As a result, more research on these promising candidate biomarkers is warranted to confirm the correlation between their single nucleotide polymorphisms and cancer risk.

The genetic variant of miR-196a2, a C to T nucleotide substitution, has been well studied and is thought be related to OSCC risk. Located in a region of the homeobox (HOX) genes, miR-196a plays an important role in regulating HOX expression and function [22]. By targeting important regulatory molecules, including HOX8 [23], miR-196 affects oncogenesis [24]. However, miR-196a either promotes or inhibits tumorigenesis, depending on the type of cancer. In breast cancer cells, pre-miR-196a2-T is converted less frequently than pre-mir-196a2-C into the mature miR-196a [25]. That finding is in agreement with our findings that the TT genotype of microRNA-196a2 may inhibit the genesis of OSCC. Furthermore, our pooled analysis was in agreement with a previous meta-analysis, which revealed that the upregulation of miR-196a2 is a biomarker that can discriminate between OSCC patients and healthy controls [26]. In the present study, considering the insignificant heterogeneity, non-publication bias and statistically significant association in the sensitivity analysis of microRNA-196, it could be concluded that the TT genotype of microRNA-196a2 can reduce OSCC risk.

For microRNA-146a, the G > C substitution within the pre-miR-146a sequence resulted in less conversion into mature miR-146a by affecting the efficiency of pri-miR-146a processing. Additionally, that substitution disturbed protein binding to pre-miR-146a [27]. Incontrovertible evidence has shown that dysregulated microRNA-146a expression is related to tumorigenesis, progression and prognosis [28]. However, the role of miR-146a is complex and controversial. Increased expression of miR-146a was suggested to be a biomarker for detecting, diagnosing and tracing cancers [29]. Although a previous meta-analysis demonstrated that the C allele of miR-146a increased the predisposition for head and neck cancer in the Caucasian population [30], our research was more reliable because it included seven eligible studies rather than three studies. A meta-analysis of microRNAs and risk of urological cancer also showed that the miR-146a G > C polymorphism reduced cancer risk [31]. Furthermore, a pooled analysis from a meta-analysis including 2 studies showed that the miR-146a polymorphism could reduce nasopharyngeal and OSCC risk but increase cervical cancer and skin squamous cell cancer risk [32]. Therefore, the miR-146a G > C polymorphism is strongly believed to be protective against OSCC.

The miR-149 polymorphism involves a C>T substitution. Through diverse signaling pathways, miR-149 targets Akt1, a gene that inhibits tumor cell apoptosis, thereby exerting its oncogenic potential [33]. No association between the miR-149 polymorphism and cancer risk has been identified in a meta-analysis of head and neck cancer susceptibility [30]. Likewise, in the present study, no significant association was discovered under any of the genetic models.

Located in the 3p mature miRNA, the miR-499 rs3746444 A/G polymorphism interferes with miRNA binding and pre-miRNA maturation [34]. Although arm selection influences cancer progression, the miR-499 polymorphism may not affect its arm selection, so it will nonetheless reduce miR-499 expression, especially miR-499-5p [19]. The miR-499a polymorphism was previously shown to be related to head-and-neck cancer and cervical squamous cell carcinoma [35]. The reason that various studies have yielded discrepancies is that they analyzed different populations or cancer locations. Furthermore, miR-499 also has varying effects on cancer risk, depending on the cancer type. Our analysis demonstrated that the miR-499 polymorphism may exert protective effects against OSCC, and this finding was consistent with the reduced expression of miR-499 in OSCC tissues, as well as the negative correlation between mir-499 expression and tumor size in clinical findings.

Many limitations might have influenced our findings. The most obvious limitation was the small number of included studies. Further research investigating microRNA polymorphisms and oral squamous cell cancer risk is warranted to confirm these findings. Furthermore, the poor consistency indicated by the sensitivity analysis of the recessive models of microRNA-196a2 and microRNA-146a might have influenced the statistical power of our study. Selection bias of included controls should be avoided by basing selection on the population rather than the hospital. Moreover, environmental factors, such as habits, should be balanced among studies. Other limitations of this study include a lack of subgroup analysis, disparity in OSCC location and the fact that we did not clarify non-response rates. Our meta-analysis failed to avoid these limitations due to a deficiency of data.

## CONCLUSIONS

To date, studies concerned with microRNA polymorphisms and the risk of oral squamous cell cancer are insufficient and inconsistent. This review focused on four microRNA polymorphisms. The results of our meta-analysis suggest that the microRNA-196a2 and microRNA-146a polymorphisms may increase OSCC risk. In contrast, the miR-499 polymorphism may exert protective effects against OSCC risk. There may be no significant association between the microRNA-149 polymorphism and OSCC risk.

Due to the small number of included studies, the associations between the selected SNPs of these four microRNAs and OSCC risk are weak.

## MATERIALS AND METHODS

### Literature sources and search strategy

The protocol details were registered with the public registry of systematic reviews, PROSPERO (CRD42017073480). Our study was conducted by following the recommended Preferred Reporting Items for Systematic Reviews and Meta-Analyses (PRISMA) statement. A search of PubMed, Embase, Web of Science and SCOPUS was conducted through June 15, 2017, to identify all the relevant studies without language limitation. The search strategy, which employed free-text words, is shown in Table 4.

**Table 4:** The search strategy for each database

Database	Search strategy
PubMed	((((((miRNA genes) OR microRNA) OR miRNA)) AND ((((((((OSCC) OR oral squamous cell carcinoma) OR mouth neoplasm) OR oral cancer) OR oral carcinoma) OR oral tumor))))) AND ((((((((((((((Variants) OR SNP) OR polymorphisms) OR mutation) OR allele) OR genotype) OR Susceptibility) OR Variant) OR SNPs) OR polymorphism) OR mutations) OR alleles) OR genotypes) OR Susceptibilities)
Embase	(exp microRNA AND (exp genetic polymorphism/ or exp single nucleotide polymorphism/ or exp DNA polymorphism) AND exp mouth squamous cell carcinoma))
Web of science	TOPIC: (miRNA) OR TOPIC: (microRNA) OR TOPIC: (microRNAs) OR TOPIC: (miRNA) AND TOPIC: (OSCC) OR TOPIC: (oral squamous cell carcinoma) OR TOPIC: (mouth neoplasm) OR TOPIC: (oral cancer) OR TOPIC: (oral carcinoma) OR TOPIC: (oral tumor) AND TOPIC: (Variants) OR TOPIC: (SNP) OR TOPIC: (polymorphism) OR TOPIC: (mutation) OR TOPIC: (allele) OR TOPIC: (genotype)
SCOPUS	((TITLE-ABS-KEY (mirna) OR TITLE-ABS-KEY (microrna) OR TITLE-ABS-KEY (micrornas) OR TITLE-ABS-KEY (mirnas))) AND ((TITLE-ABS-KEY (oscc) OR TITLE-ABS-KEY (oral AND squamous AND cell AND carcinoma) OR TITLE-ABS-KEY (mouth AND neoplasm) OR TITLE-ABS-KEY (oral AND cancer) OR TITLE-ABS-KEY (oral AND carcinoma) OR TITLE-ABS-KEY (oral AND tumor))) AND ((TITLE-ABS-KEY (variants) OR TITLE-ABS-KEY (snp) OR TITLE-ABS-KEY (polymorphisms) OR TITLE-ABS-KEY (mutation) OR TITLE-ABS-KEY (allele) OR TITLE-ABS-KEY (genotype)))

Next, titles and abstracts were read manually for preliminary analysis, in duplicate, by two reviewers. Disagreement was resolved by discussion or by referring the debate to a third reviewer. Finally, the chosen articles were subjected to the following inclusion and exclusion criteria:

### Selection criteria

1. The included articles should be case-controls. 2. Patients with a definite diagnosis of oral cancer, not oral precancer, should be mentioned. 3. The included studies must contain extractable data on SNPs in pre-miR distribution in cases and controls.

### Exclusion criteria

1. Articles are review articles or they describe experiments in animals. 2. Studies focus on miRNA-processing genes or miRNA-binding site genes instead of pre-miRNA genes. 3. Studies report non-extractable data. 4. Studies in which the *P* value for HWE (*P*_HWE_) in the control group was less than 0.05 would be eliminated from the sensitivity analysis. 5. Studies are repeated studies by the same author or team.

### Quality assessment and data extraction

Each chosen study was subjected to thorough reading for data extraction, synthesis, analysis and quality assessment, following the Newcastle-Ottawa Scale (NOS) system (http://www.ohri.ca/programs/clinical_epidemiology/oxford.asp) independently and strictly.

### Outcome variables and data analysis

The minor allele frequency (MAF) was confirmed in PubMed. The outcome variables consisted of the allele and genotype frequencies of miRNA polymorphisms in cases and controls. Stata version 14.0 software was used for the data analysis. The odds ratio (OR) and 95% confidence interval were calculated for five genetic models: the allele model, the additive model, the codominant model, the dominant model and the recessive model. The *I*^*2*^ test was chosen to test for statistical heterogeneity. If I^2^ ≥ 50%, which indicates substantial heterogeneity, we used a random-effects model to summarize outcome variables. If I^2^ < 50%. meta-analyses were reported using a fixed-effects model. The hypothesis tested was defined as statistically significant when the *P* value was < 0.05.

## Abbreviations

OSCC: oral squamous cell cancer
ORs: Odds ratios
miRNAs: microRNAs
UTR: untranslated region
SNPs: single nucleotide polymorphisms
HWE: Hardy-Weinberg equilibrium
HOX: homeobox
PROSPERO: public registry of systematic reviews
PRISMA: Preferred Reporting Items for Systematic Reviews and Meta-Analyses
NOS: Newcastle-Ottawa Scale
MAF: Minor allele frequency
